# Validity of a low-cost friction encoder for measuring velocity, force and power in flywheel exercise devices

**DOI:** 10.5114/biolsport.2023.119991

**Published:** 2022-11-18

**Authors:** Víctor Illera-Domínguez, Bruno Fernández-Valdés, Jose Gisbert-Orozco, Carlos Ramirez-Lopez, Sergi Nuell, Jacob González, Jonathon Weakley

**Affiliations:** 1 Institut Nacional d’Educació Física de Catalunya (INEFC), Universitat de Barcelona, Barcelona, Spain; 2 Tecnocampus, Department of Health Sciences, Pompeu Fabra University, Mataró, Spain; 3 Carnegie Applied Rugby Research (CARR) centre, Institute for Sport, Physical Activity and Leisure, Carnegie School of Sport, Leeds Beckett University, Leeds, United Kingdom; 4 Scottish Rugby Union, Murrayfield Stadium, Edinburgh, United Kingdom; 5 Futbol Club Barcelona (FCB), Barcelona, Spain; 6 School of behavioral and health sciences, Australian Catholic University, Brisbane, Australia

**Keywords:** Iso-inertial, Monitoring, Resistance Training, Feedback

## Abstract

The purpose of this study was to investigate the validity of a low-cost friction encoder against a criterion measure (strain gauge combined with a linear encoder) for assessing velocity, force and power in flywheel exercise devices. Ten young and physically active volunteers performed two sets of 14 maximal squats on a flywheel inertial device (YoYo Technology, Stockholm, Sweden) with five minutes rest between each set. Two different resistances were used (0.075 kg · m^2^ for the first set; 0.025 kg · m^2^ for the second). Mean velocity (V_rep_), force (F_rep_) and power (P_rep_) for each repetition were assessed simultaneously via a friction encoder (Chronojump, Barcelona, Spain), and with a strain gauge combined with a linear encoder (MuscleLab 6000, Ergotest Technology, Porsgrunn, Norway). Results are displayed as (Mean [CI 90%]). Compared to criterion measures, mean bias for the practical measures of V_rep_, F_rep_ and P_rep_ were moderate (-0.95 [-0.99 to -0.92]), small (0.53 [0.50 to 0.56]) and moderate (-0.68 [-0.71 to -0.65]) respectively. The typical error of estimate (TEE) was small for all three parameters; V_rep_ (0.23 [0.20 to 0.25]), F_rep_ (0.20 [0.18 to 0.22]) and P_rep_ (0.18 [0.16 to 0.20]). Correlations with MuscleLab were nearly perfect for all measures in all load configurations. Based on these findings, the friction encoder provides valid measures of velocity, force and power in flywheel exercise devices. However, as error did exist between measures, the same testing protocol should be used when assessing changes in these parameters over time, or when aiming to perform inter-individual comparisons.

## INTRODUCTION

Quantifying training loads is an important consideration for sports scientists and practitioners. Accurate quantification can ensure that training is both appropriate and meets the programme requirements [1]. Furthermore, this information is essential for informing decision making so that negative training outcomes (e.g. overtraining, illness, and maladaptation) can be avoided [2]. For this reason, tools that are used to quantify training need to be valid so that accurate training information can be gathered. This is relevant for all training methods, including resistance training.

Traditional resistance training methods (e.g. gravity-dependent loads such as dumbbells and barbells), are often quantified through the mass and number of repetitions completed (e.g. volume load) [3]. However, alternative methods of resistance training, such as flywheel training, can be more challenging to quantify as they do not have a constant external load and rely on athletes overcoming inertia to accelerate or decelerate the mass of the wheels (i.e. isoinertial training) [4]. Despite these practical difficulties in quantifying training loads, the popularity of flywheel devices has grown in recent times due to their ability to induce positive adaptations in strength, power, and velocity [5]. Furthermore, their ability to easily apply eccentric overload (i.e. brief episodes in which eccentric output is higher compared to the concentric output) may provide benefits that can be difficult to achieve using traditional training methods [5, 6]. For an extended description of the eccentric overload concept, relevance, and the available ways for its quantification the reader is referred to a recent review on the topic [7].

Several laboratory technologies can be used for quantifying movement velocity (e.g. linear encoders or optical motion sensing systems) and force (e.g. force plates or strain gauges) during isoinertial exercises [8–12]. However, since some of the aforementioned tools are expensive laboratory-based pieces of equipment, these are not viable options for most practitioners. Another limitation is that power measurement requires the combination of at least two sensors for being calculated as the product of force and velocity [9, 10]. Therefore, the necessary set-up of sensors and wires may not be convenient in day-to-day training.

To facilitate the quantification of isoinertial training loads, monitoring devices known as axis rotary encoders have been developed. These kinetic sensors are welded to the axis to track flywheel angular velocity and relate these data to a constant moment of inertia to estimate applied force and power in real time. Recent works have demonstrated that flywheel resistance training can be accurately quantified through the use of these sensors [9, 10, 13]. However, one of the biggest limitations is that axis rotary encoders have to be installed by the manufacturer through industrial mechanization of the pieces. In this sense, the encoder is specific to the isoinertial training system and can not be disarmed and used with different devices.

To circumnavigate this issue, rotary friction encoders have been developed. These sensors are not specific or welded to a unique isoinertial training system, but instead can be adapted and used to monitor different isoinertial machines. This is a very relevant feature, as it allows for monitoring existing non-sensorized equipment in a few minutes. Rotary friction encoders consist of a wheel which has to be tightly attached to the rotating flywheel. Due to friction between the two surfaces, the encoder shares the same linear velocity with the point where it is installed. Force, power, and velocity that is being produced by the athlete during training are calculated in real time from the encoder measures of rotational velocity, using given configuration parameters such as the moment of inertia of the wheel, additional masses, and diameters of the pieces (e.g. axis). Therefore, by introducing these set-up parameters in the software, the sensor can be adapted to different inertial training systems.

Although the practicality and relevance of rotary friction encoders is evident, their criterion validity has not been assessed, so the accuracy of the provided feedback is still unknown. Thus, the aim of this study was to assess the criterion validity of force, power, and velocity outputs that are calculated at two different isoinertial loads from a rotary friction encoder.

## MATERIALS AND METHODS

### Experimental Approach to the Problem

The study was designed to evaluate the criterion validity of force, velocity and power values measured with a commonly used low-cost friction encoder (Chronojump, Barcelona, Spain). In order to do so, half-squats performed on a flywheel with two different isoinertial loads (0.075 kg · m^2^ and 0.025 kg · m^2^) were simultaneously monitored using (I, practical measure) a friction encoder attached at a known diameter of the flywheel, and (II, criterion measure) a strain gauge combined with a linear encoder attached to the harness. The level of agreement between force, velocity and power obtained from both methods was assessed.

### Subjects

Ten physically active male and female volunteers (age 27.9 ± 4.9 years, body mass 70.6 ± 11.9 kg, height 174.5 ± 8.3 cm) were recruited to take part in this study. Subjects were free of injury and illness during data collection. All subjects had previously trained with the exercise device and were familiar with the testing protocol and isoinertial loads used. All experimental procedures were approved by the Catalan Sports Council (Generalitat de Catalunya) ethics committee, and written consent was provided by all subjects before study initiation.

### Procedures

An exercise protocol consisting of maximal voluntary concentric-eccentric bilateral half-squats was performed on a “YoYo squat” flywheel device (YoYo Technology, Stockholm, Sweden). An extended description of the exercise can be found elsewhere [14]. Subjects performed two sets of the exercise, consisting of three sub-maximal reps to accelerate the disk, followed by 14 repetitions with maximal intent. In order to test the validity of the friction encoder in different load configurations, two different resistances were used: The moment of inertia was 0.075 kg · m^2^ for one set and 0.025 kg · m^2^ for the other. These load configurations were selected because they are commonly used both in research and in the professional practice [6, 7] and have previously been described as high and low isoinertial load configurations for the squat [15]. The order that subjects completed these different isoinertial loads were randomized by a coin toss. Mean velocity (V_rep_), force (F_rep_) and power (P_rep_) for each repetition were assessed simultaneously via a friction encoder, and strain gauge combined with a linear encoder.

**FIG. 1:**
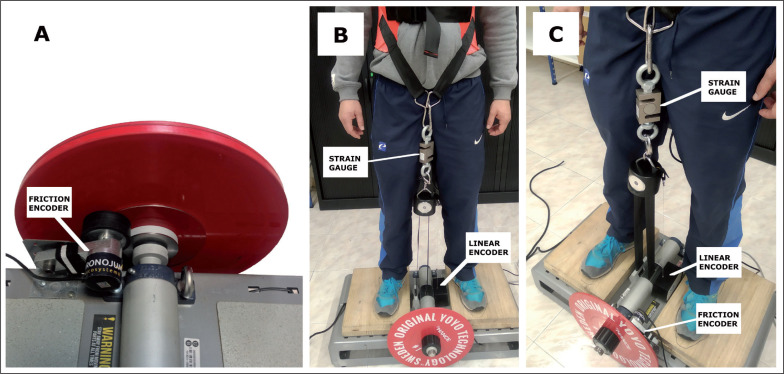
Disposition of the different sensors used in the study. (A) The friction encoder was tightly attached at a known diameter of the flywheel, sharing the same linear velocity. (B) The strain gauge was attached between the strap of the flywheel and the vest, and the linear encoder was placed on the flywheel device and attached to the vest, forming a perpendicular angle with the floor. (C) General view of the setup.

Criterion measures of velocity, force and power were assessed by a strain gauge synchronized with a linear encoder using a Muscle-Lab 6000 (Ergotest Technology, Porsgrunn, Norway). This device has been used before as a measurement reference system in inertial training [10, 12]. The strain gauge (accuracy 63 g, sampling rate: 200 Hz) was attached between the strap of the flywheel and the vest; and the linear encoder (accuracy 0.019 mm; sampling rate 200 Hz) was placed on the flywheel device and attached to the vest, forming a perpendicular angle with the floor (Figure 1).

The friction encoder (Chronojump, Barcelona, Spain), (accuracy 1 mm, sampling rate 1000 Hz) was tightly attached at a known diameter of the flywheel, sharing the same linear velocity (Figure 1). The set-up parameters such as disk inertia, axis diameter, and the volunteer’s body mass were entered into the associated Chronojump software (v1.8.1–95) to compute the practical measures of velocity, force and power in real time. Chronojump is an open-code software, and a complete repository of the code and formulas used can be found online [16].

As previously introduced, axis diameter is an essential parameter for computing kinematic variables from the friction encoder, because it determines the point at which the strap exerts unwinding force, and therefore modifies the moment arm and torque. In the particular training device used in this study, the diameter is slightly variable across the repetition, given that the strap rolls over on itself increasing the diameter. For this reason, the mean diameter value was used for the estimations.

Raw data from each of the 14 high-intensity repetitions of each set were averaged to compute repetition mean velocity (V_rep_), force (F_rep_) and power (P_rep_), for both measuring systems.

### Statistical Analysis

Agreement between the criterion measures (MuscleLab) and the practical measures (Chronojump) of V_rep_, F_rep_ and P_rep_ was assessed using an excel spreadsheet for analysis of validity [17] designed to calculate the mean bias, typical error of the estimate (TEE) and Pearson correlation coefficient, all with 90% confidence limits. The standardised mean bias was rated as trivial (≤ 0.19), small (0.2–0.59), moderate (0.6–1.19) or large (1.2–1.99) [17]. The standardised typical error was rated as trivial (< 0.1), small (0.1–0.29), moderate (0.3–0.59) or large (> 0.59) [17]. The magnitude of correlation was rated as trivial (< 0.1), small (0.1–0.29), moderate (0.3–0.49), large (0.5–0.69), very large (0.7–0.89) or nearly perfect (0.9–0.99) [17]. Additionally, Bland–Altman plots were used to graphically complement the differences between the two systems [18].

## RESULTS

When compared to criterion measures, mean bias for the practical measures of V_rep_, F_rep_ and P_rep_ were moderate, small and moderate respectively. The TEE was small for all three parameters (Table 1). Correlations with MuscleLab were nearly perfect for all measures in all load configurations (Table 1).

**TABLE 1:** Comparison of V_rep_, F_rep_ and P_rep_ between MuscleLab and Chronojump at high (0.075 kg · m^2^) and low (0.025 kg · m^2^) load configurations, and the total pooled data. Data is presented as mean values (± standard deviation (SD)) and mean bias, typical error of the estimate and Pearson correlation coefficient, all with 90% confidence limits.

Load	Variable	Criterion Measure	Practical Measure	Bias	TEE	Correlation
High (0.075 kg · m^2^)	V_rep_	MuscleLab	Chronojump	-1.44 [-1.49 to -1.39]	0.26 [0.23 to 0.31]	0.97 [0.96 to 0.97]
		0.37 ± 0.06 m/s	0.28 ± 0.04 m/s	(*large*)	(*small*)	(*nearly perfect*)
	F_rep_	MuscleLab	Chronojump	0.67 [0.63 to 0.70]	0.18 [0.15 to 0.20]	0.98 [0.98 to 0.99]
		414.2 ± 160.6 W	312.4 ± 118.2 W	(*moderate*)	(*small*)	((*nearly perfect*)
	P_rep_	MuscleLab	Chronojump	-0.63 [-0.67 to -0.59]	0.13 [0.12 to 0.15]	0.99 [0.99 to 0.99]
		414.2 ± 160.6 W	312.4 ± 118.2 W	(*moderate*)	(*small*)	(*nearly perfect*)

Low (0.025 kg · m^2^)	V_rep_	MuscleLab	Chronojump	-1.48 [-1.54 to -1.42]	0.38 [0.33 to 0.44]	0.93 [0.91 to 0.95]
		0.55 ± 0.10 m/s	0.41 ± 0.07 m/s	(*large*)	(*moderate*)	(*nearly perfect*)
	F_rep_	MuscleLab	Chronojump	0.50 [0.46 to 0.54]	0.27 [0.24 to 0.32]	0.96 [0.95 to 0.97]
		830.1 ± 222.3 N	942.0 ± 243.9 N	(*small*)	(*small*)	(*nearly perfect*)
	P_rep_	MuscleLab	Chronojump	-0.75 [-0.80 to -0.70]	0.20 [0.17 to 0.23]	0.98 [0.97 to 0.99]
		478.0 ± 177.9 W	344.8 ± 126.5 W	(*moderate*)	(*small*)	(*nearly perfect*)

Total	V_rep_	MuscleLab	Chronojump	-0.95 [-0.99 to -0.92]	0.23 [0.20 to 0.25]	0.98 [0.97 to 0.98]
		0.46 ± 0.13 m/s	0.34 ± 0.09 m/s	(*moderate*)	(*small*)	(*nearly perfect*)
	F_rep_	MuscleLab	Chronojump	0.53 [0.50 to 0.56]	0.20 [0.18 to 0.22]	0.98 [0.98 to 0.98]
		960.1 ± 297.1 N	1118.3 ± 350.7 N	(*small*)	(*small*)	(*nearly perfect*)
	P_rep_	MuscleLab	Chronojump	-0.68 [-0.71 to -0.65]	0.18 [0.16 to 0.20]	0.98 [0.98 to 0.99]
		446.1 ± 172.2 W	328.6 ± 123.3 W	(moderate)	(*small*)	(*nearly perfect*)

V_rep_ = Mean repetition velocity. F_rep_ = Mean repetition force. P_rep_ = Mean repetition power

**FIG. 2:**
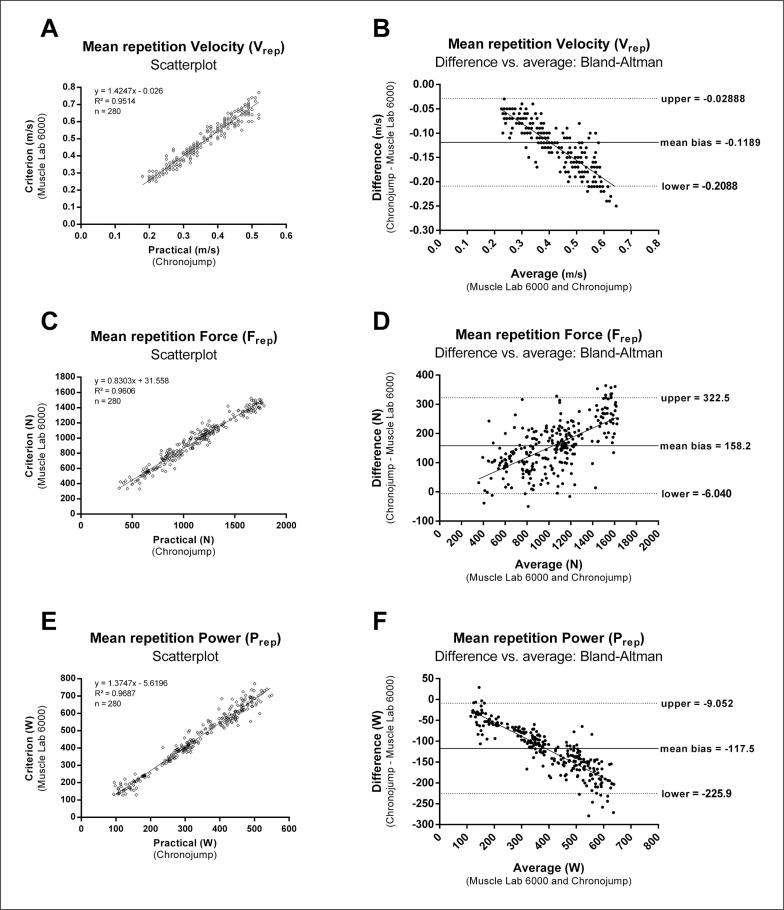
Scatterplots showing the agreement between the criterion and practical measures V_rep_ (A), F_rep_ (C) and P_rep_ (E). Bland-Altman plots for V_rep_ (B), F_rep_ (D) and P_rep_ (F). The solid horizontal line within the graphs represents the mean bias. The broken horizontal lines represent the upper and lower 95% limits of agreement. The regression lines of the scattered points are shown to investigate the homoscedasticity of the errors.

The regression equations to estimate the criterion measures from the practical measures fitted well with data (Figure 2A, 2C, 2E) and are presented as follows:

Y=intercept+(slope⋅X)

where Y is the estimated criterion measure and X is the practical measure.

The regression equation of V_rep_ is:

Y=−0.026+(1.4247⋅X)

The regression equation of F_rep_ is:

Y=31.558+(0.8303⋅X)

The regression equation of P_rep_ is:

Y=−5.6196+(1.3747⋅X)

The Bland–Altman plots visually showed the differences between the two systems for the measurement of V_rep_, F_rep_ and P_rep_ (Figure 2B, 2D, 2F).

## DISCUSSION

The primary finding of this study demonstrates acceptable levels of agreement between a low-cost, easy and versatile method (Chronojump friction encoder) and criterion measure (strain gauge combined with a linear encoder) for the assessment of velocity, force and power in a flywheel exercise device with different isoinertial loads. The friction encoder demonstrated small to moderate TEE and nearly perfect relationships across all kinetic and kinematic variables at both isoinertial loads. However, small to large bias was demonstrated, which should be acknowledged when implemented in training and future research. Considering these findings, the Chronojump friction encoder is a valid tool for the quantification of training loads when completing flywheel resistance training.

The monitoring of kinetic and kinematic variables is an important consideration for the accurate quantification of resistance training intensity. Furthermore, these external stimuli provide a greater understanding of the internal and fatigue responses that determine muscular adaptations [19]. Small to moderate TEE for all were found, which suggests that the Chronojump device can adequately provide repetition information during exercise. This will not only be of use to practitioners and scientists but also athletes, as this device can provide live augmented feedback that may enhance psychological traits during resistance training and subsequent physical adaptations [20]. However, the small to large bias that was present suggests that findings may be smaller or greater depending on the variable assessed (i.e. velocity and power may be consistently lower and force consistently higher than actual values). Thus, it is advised that, due to the lowest TEE and bias observed, power outputs are monitored in the detection of changes in neuromuscular performance.

Differences between measurement systems have also shown to be proportional to the magnitude of the value measured for all three parameters assessed, as indicated by the regression equations (see figure 2A, 2C, 2E) and the slope of the regression lines in Bland Altman plots (see figure 2B, 2D, 2F) [21]. Therefore, lower absolute bias is to be expected when the magnitude of applied V_rep_, F_rep_ or P_rep_ are low.

One limitation of this study is that measurements have been exclusively registered from one inertial training system (“YoYo squat” – YoYo Technology, Stockholm, Sweden). Therefore, the extrapolation of the results to substantially different systems (i.e. conic pulleys) is limited. Another aspect to take into account is that the diameter of the axis of the system used was slightly variable across the range of motion, depending on the winding up of the strap (i.e. as the stap winds up over deeper layers of the strap, the diameter increases). Therefore, the mean diameter of the axis was used for the calculations. As this fact may have contributed to increasing the error of measurement, it is to be expected better accuracy in other inertial systems in which axis diameter remains constant throughout the repetition.

Despite the existence of other valid methods to monitor velocity, force and power in flywheel devices [9, 10], to our knowledge currently there are no other low-cost tools that work with free, open-code software to monitor a wide variety of flywheel resistance exercises. Given the practicality (e.g. minimal set-up), versatility (e.g. applicable to multiple systems and configurations) and cost of this sensor compared with other available options, practitioners may prefer this method for assessing V_rep_, F_rep_ and P_rep_ in flywheel exercises.

## CONCLUSIONS

In the last years, there has been a growing interest in flywheel resistance training [22]. Research in this area has shown greater increases in power and force with flywheel training [6, 23–25], improvements in changes of directions [26, 27] and other kinematics responses [28, 29], early hypertrophic adaptations [14] and changes in movement variability with functional resistance training exercises [30]. Nonetheless, only a few works have investigated the accuracy of rotary encoders for monitoring flywheel resistance training [9, 10, 13], and none had validated the use of rotary friction encoders, which can be adapted to different inertial training systems.

Inasmuch as the importance of the use of feedback [20, 31] and the control of velocity loss [32, 33] in resistance training using free weights, the validation of this rotary friction encoder and his opencode software could help strength and conditioning coaches to confidently monitor force, power and velocity in any flywheel machine, and therefore improve the control of training whenever using these resistance training devices, both with low and high training loads.

Coaches and sports scientists may confidently use a Chronojump friction encoder for assessing velocity, force and power in flywheel exercises, allowing for a simplified, accurate and versatile way of testing and monitoring athletes daily.
